# 3-{2-[(1,3-Benzothia­zol-2-yl)sulfanyl­meth­yl]phen­yl}-4-meth­oxy-5,5-dimethyl­furan-2(5*H*)-one

**DOI:** 10.1107/S1600536812022404

**Published:** 2012-05-23

**Authors:** Anna Duan, Haikui Yang, Peiliang Zhao, Wenwei You

**Affiliations:** a School of Pharmaceutical Sciences, Southern Medical University, Guangzhou 510515, People’s Republic of China

## Abstract

In the title compound, C_21_H_19_NO_3_S_2_, the dihedral angles formed between the thia­zole ring and the adjacent benzene ring and the other benzene ring are 1.58 (3) and 76.48 (6)°, respectively. The crystal structure features a weak C—H⋯O inter­action.

## Related literature

For the anti-tumor activity of benzothia­zole derivatives, see: Brantley *et al.* (2004 ▶) and for their anti-tuberculous properties, see: Palmer *et al.* (1971 ▶). For fungicidal properties of benzothia­zolines and the preparation of the title compound, see: Zhao *et al.* (2010 ▶). For general background to furan-2(5*H*)-ones and their derivatives, see: Iannazzo *et al.* (2008 ▶).
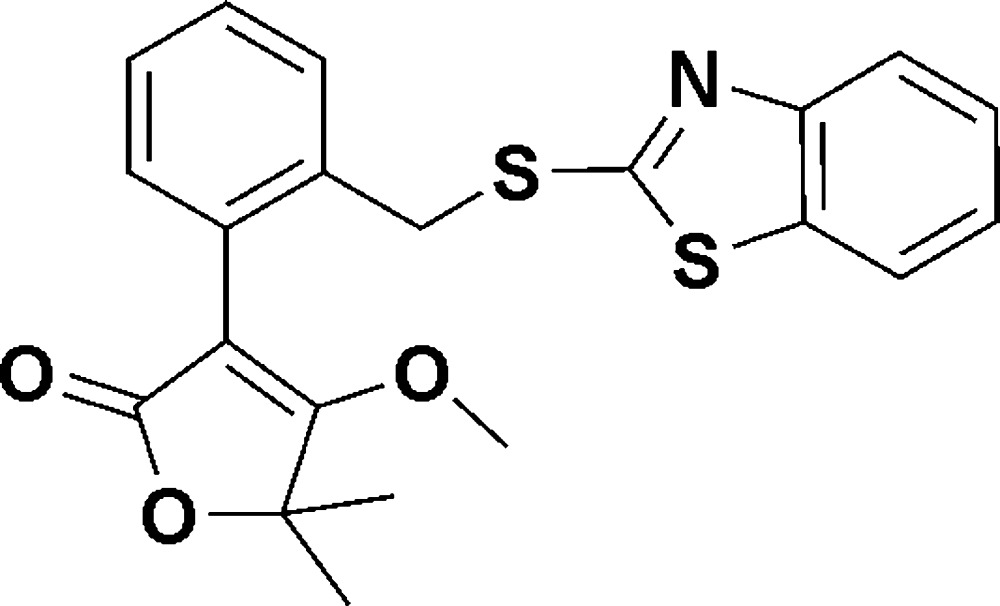


## Experimental

### 

#### Crystal data

C_21_H_19_NO_3_S_2_
*M* *_r_* = 397.49
Triclinic, 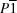

*a* = 10.4702 (9) Å
*b* = 10.4851 (9) Å
*c* = 10.5169 (9) Å
α = 117.567 (1)°
β = 100.398 (1)°
γ = 95.257 (1)°
*V* = 986.15 (15) Å^3^
*Z* = 2
Mo *K*α radiation
μ = 0.29 mm^−1^
*T* = 292 K
0.20 × 0.20 × 0.10 mm

#### Data collection

Bruker SMART CCD area-detector diffractometer
Absorption correction: multi-scan (*SADABS*; Sheldrick, 2005 ▶) *T*_min_ = 0.944, *T*_max_ = 0.972
7206 measured reflections
3804 independent reflections
2713 reflections with *I* > 2σ(*I*)
*R*_int_ = 0.040

#### Refinement

*R*[*F* ^2^ > 2σ(*F* ^2^)] = 0.045
*wR*(*F* ^2^) = 0.109
*S* = 0.90
3804 reflections
247 parameters
H-atom parameters constrained
Δρ_max_ = 0.25 e Å^−3^
Δρ_min_ = −0.22 e Å^−3^

### 

Data collection: *SMART* (Bruker, 2001 ▶); cell refinement: *SAINT* (Bruker, 2001 ▶); data reduction: *SAINT*; program(s) used to solve structure: *SHELXS97* (Sheldrick, 2008 ▶); program(s) used to refine structure: *SHELXL97* (Sheldrick, 2008 ▶); molecular graphics: *SHELXTL* (Sheldrick, 2008 ▶); software used to prepare material for publication: *SHELXTL*.

## Supplementary Material

Crystal structure: contains datablock(s) global, I. DOI: 10.1107/S1600536812022404/im2376sup1.cif

Structure factors: contains datablock(s) I. DOI: 10.1107/S1600536812022404/im2376Isup2.hkl

Supplementary material file. DOI: 10.1107/S1600536812022404/im2376Isup3.cml

Additional supplementary materials:  crystallographic information; 3D view; checkCIF report

## Figures and Tables

**Table 1:** Hydrogen-bond geometry (Å, °)

*D*—H⋯*A*	*D*—H	H⋯*A*	*D*⋯*A*	*D*—H⋯*A*
C2—H2⋯O1^i^	0.93	2.56	3.302 (3)	137

Symmetry code: (i) 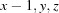.

**Fig. 1.**
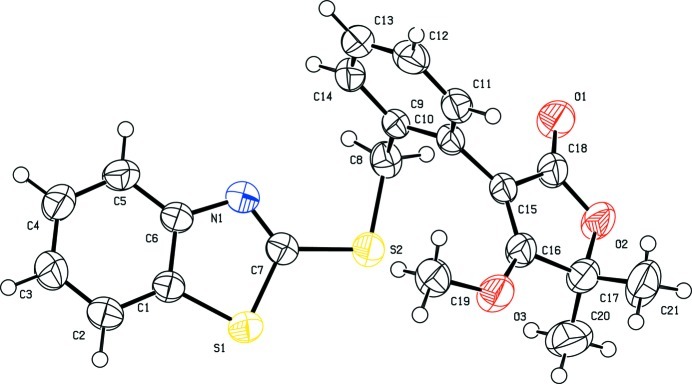
A view of the molecule of (I) showing displacement ellipsoids at the 50% probability level. H atoms are represented by circles of arbitrary size.

**Fig. 2.**
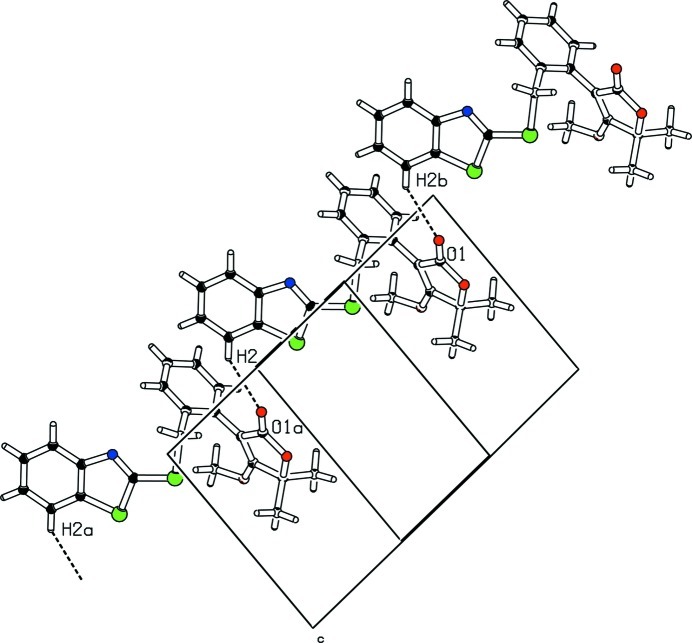
Hydrogen bonding in the crystal structure of (I). Hydrogen bonds are shown as dashed lines. [Symmetry codes: x - 1, y, z]

## supplementary crystallographic information

### Comment

Benzothiazole derivatives are well known to exhibit a wide spectrum of biological activities, including fungicidal, anticancer and anti-tuberculous properties. (Brantley *et al.*, 2004; Zhao *et al.*, 2010; Palmer *et al.*, 1971). In addition, furan-2(5*H*)-one derivatives also have good biological activities (Iannazzo *et al.*, 2008). These findings prompted us to synthesize a new series of benzothiazole derivatives by incorporating furan-2(5*H*)-one at the 2-position, in the hope of finding molecules showing an improved bioactivity. We present here the X-ray crystallographic analysis of the title compound, (I), which was designed and synthesized in our laboratory.

A view of the molecular structure of the title compound is given in Fig.1. The bond lengths and angles are unremarkable.The dihedral angles formed between the triazole ring and the adjacent benzene ring and the other benzene ring system are 1.58 (3)° and 76.48 (6)°, respectively. One intermolecular C—H···O hydrogen bond exists in the crystal structure (Table 1). Atom C2 in the molecule acts as donor, *via* the H atom H2, towards O1 of an adjacent molecule (Fig.2). No π-π-stacking interactions are observed in the crystal structure.

### Experimental

The title compound was synthesized according to a published procedure (Zhao *et al.*, 2010). Crystals appropriate for X-ray data collection were obtained by slow evaporation of a methanolic solution at 292 K.

### Refinement

All H atoms were initially located in a difference Fourier map. Methyl H atoms were then constrained to an ideal geometry with C—H distances of 0.96 Å and *U*_iso_(H) = 1.5*U*_eq_(C), but each group was allowed to rotate freely about its C—C bond. All other H atoms were placed in geometrically idealized positions and constrained to ride on their parent atoms with C—H distances in the range 0.93–0.97 Å and *U*_iso_(H) = 1.2*U*_eq_(C).

### Crystal data

**Table:**

C_21_H_19_NO_3_S_2_	*Z* = 2
*M**_r_* = 397.49	*F*(000) = 416
Triclinic, *P*1	*D*_x_ = 1.339 Mg m^−^^3^
Hall symbol: -P 1	Mo *K*α radiation, λ = 0.71073 Å
*a* = 10.4702 (9) Å	Cell parameters from 2192 reflections
*b* = 10.4851 (9) Å	θ = 2.3–24.6°
*c* = 10.5169 (9) Å	µ = 0.29 mm^−^^1^
α = 117.567 (1)°	*T* = 292 K
β = 100.398 (1)°	Block, colorless
γ = 95.257 (1)°	0.20 × 0.20 × 0.10 mm
*V* = 986.15 (15) Å^3^	

### Data collection

**Table:**

Bruker SMART CCD area-detector diffractometer	3804 independent reflections
Radiation source: fine-focus sealed tube	2713 reflections with *I* > 2σ(*I*)
Graphite monochromator	*R*_int_ = 0.040
phi and ω scans	θ_max_ = 26.0°, θ_min_ = 2.0°
Absorption correction: multi-scan (*SADABS*; Sheldrick, 2005)	*h* = −11→12
*T*_min_ = 0.944, *T*_max_ = 0.972	*k* = −12→12
7206 measured reflections	*l* = −12→12

### Refinement

**Table:**

Refinement on *F*^2^	Primary atom site location: structure-invariant direct methods
Least-squares matrix: full	Secondary atom site location: difference Fourier map
*R*[*F*^2^ > 2σ(*F*^2^)] = 0.045	Hydrogen site location: inferred from neighbouring sites
*wR*(*F*^2^) = 0.109	H-atom parameters constrained
*S* = 0.90	*w* = 1/[σ^2^(*F*_o_^2^) + (0.0592*P*)^2^] where *P* = (*F*_o_^2^ + 2*F*_c_^2^)/3
3804 reflections	(Δ/σ)_max_ < 0.001
247 parameters	Δρ_max_ = 0.25 e Å^−^^3^
0 restraints	Δρ_min_ = −0.22 e Å^−^^3^

### Special details

**Table:**

Geometry. All e.s.d.'s (except the e.s.d. in the dihedral angle between two l.s. planes) are estimated using the full covariance matrix. The cell e.s.d.'s are taken into account individually in the estimation of e.s.d.'s in distances, angles and torsion angles; correlations between e.s.d.'s in cell parameters are only used when they are defined by crystal symmetry. An approximate (isotropic) treatment of cell e.s.d.'s is used for estimating e.s.d.'s involving l.s. planes.
Refinement. Refinement of *F*^2^ against ALL reflections. The weighted *R*-factor *wR* and goodness of fit *S* are based on *F*^2^, conventional *R*-factors *R* are based on *F*, with *F* set to zero for negative *F*^2^. The threshold expression of *F*^2^ > σ(*F*^2^) is used only for calculating *R*-factors(gt) *etc*. and is not relevant to the choice of reflections for refinement. *R*-factors based on *F*^2^ are statistically about twice as large as those based on *F*, and *R*- factors based on ALL data will be even larger.

### Fractional atomic coordinates and isotropic or equivalent isotropic displacement parameters (Å^2^)

**Table:**

	*x*	*y*	*z*	*U*_iso_*/*U*_eq_	
C1	0.3099 (2)	1.1296 (2)	0.3804 (2)	0.0429 (5)	
C2	0.1915 (2)	1.1787 (3)	0.3934 (3)	0.0585 (6)	
H2	0.1101	1.1139	0.3439	0.070*	
C3	0.1987 (3)	1.3256 (3)	0.4814 (3)	0.0749 (8)	
H3	0.1207	1.3609	0.4920	0.090*	
C4	0.3186 (3)	1.4226 (3)	0.5548 (3)	0.0720 (8)	
H4	0.3199	1.5220	0.6133	0.086*	
C5	0.4367 (2)	1.3757 (2)	0.5435 (3)	0.0548 (6)	
H5	0.5172	1.4418	0.5944	0.066*	
C6	0.4325 (2)	1.2275 (2)	0.4545 (2)	0.0401 (5)	
C7	0.5064 (2)	1.0231 (2)	0.3504 (2)	0.0393 (5)	
C8	0.7722 (2)	1.0145 (2)	0.3745 (2)	0.0471 (5)	
H8A	0.7754	1.0955	0.4713	0.057*	
H8B	0.8379	0.9594	0.3878	0.057*	
C9	0.80777 (19)	1.0755 (2)	0.2782 (2)	0.0383 (5)	
C10	0.82367 (19)	0.9825 (2)	0.1364 (2)	0.0388 (5)	
C11	0.8548 (2)	1.0439 (2)	0.0510 (2)	0.0468 (5)	
H11	0.8650	0.9830	−0.0432	0.056*	
C12	0.8711 (2)	1.1929 (3)	0.1022 (3)	0.0532 (6)	
H12	0.8919	1.2318	0.0433	0.064*	
C13	0.8561 (2)	1.2839 (2)	0.2417 (3)	0.0547 (6)	
H13	0.8674	1.3847	0.2775	0.066*	
C14	0.8244 (2)	1.2252 (2)	0.3280 (2)	0.0486 (6)	
H14	0.8140	1.2873	0.4217	0.058*	
C15	0.8151 (2)	0.8231 (2)	0.0802 (2)	0.0406 (5)	
C16	0.7292 (2)	0.7023 (2)	−0.0266 (2)	0.0495 (6)	
C17	0.7745 (2)	0.5641 (3)	−0.0455 (3)	0.0630 (7)	
C18	0.9225 (2)	0.7703 (3)	0.1412 (3)	0.0514 (6)	
C19	0.5531 (2)	0.8016 (3)	−0.1034 (3)	0.0575 (6)	
H19A	0.5432	0.8556	−0.0043	0.086*	
H19B	0.4677	0.7665	−0.1725	0.086*	
H19C	0.6086	0.8647	−0.1235	0.086*	
C20	0.6809 (3)	0.4717 (3)	−0.0114 (4)	0.0940 (10)	
H20A	0.7197	0.3923	−0.0111	0.141*	
H20B	0.5983	0.4328	−0.0856	0.141*	
H20C	0.6657	0.5315	0.0841	0.141*	
C21	0.8059 (3)	0.4778 (3)	−0.1948 (3)	0.0900 (10)	
H21A	0.8612	0.5429	−0.2127	0.135*	
H21B	0.7250	0.4334	−0.2718	0.135*	
H21C	0.8515	0.4026	−0.1941	0.135*	
N1	0.54360 (17)	1.16332 (19)	0.43437 (18)	0.0421 (4)	
O1	1.02066 (17)	0.84009 (18)	0.2398 (2)	0.0706 (5)	
O2	0.89788 (16)	0.62095 (17)	0.06858 (18)	0.0649 (5)	
O3	0.61278 (16)	0.67939 (17)	−0.11829 (18)	0.0677 (5)	
S1	0.33515 (6)	0.95239 (6)	0.28478 (6)	0.04817 (18)	
S2	0.60881 (6)	0.89569 (6)	0.29888 (6)	0.04876 (19)	

### Atomic displacement parameters (Å^2^)

**Table:**

	*U*^11^	*U*^22^	*U*^33^	*U*^12^	*U*^13^	*U*^23^
C1	0.0394 (13)	0.0446 (13)	0.0388 (11)	0.0066 (10)	0.0056 (10)	0.0175 (10)
C2	0.0378 (14)	0.0557 (16)	0.0604 (15)	0.0056 (12)	0.0000 (12)	0.0162 (13)
C3	0.0425 (16)	0.0631 (18)	0.091 (2)	0.0176 (14)	0.0086 (15)	0.0167 (16)
C4	0.0551 (18)	0.0436 (15)	0.088 (2)	0.0149 (13)	0.0073 (15)	0.0121 (15)
C5	0.0463 (15)	0.0416 (14)	0.0616 (15)	0.0026 (11)	0.0019 (12)	0.0184 (12)
C6	0.0366 (12)	0.0418 (13)	0.0405 (11)	0.0050 (10)	0.0064 (10)	0.0209 (10)
C7	0.0413 (13)	0.0427 (13)	0.0372 (11)	0.0079 (10)	0.0111 (10)	0.0219 (10)
C8	0.0421 (13)	0.0541 (14)	0.0441 (12)	0.0157 (11)	0.0110 (11)	0.0221 (11)
C9	0.0265 (11)	0.0390 (12)	0.0429 (12)	0.0082 (9)	0.0058 (9)	0.0156 (10)
C10	0.0283 (11)	0.0409 (12)	0.0411 (11)	0.0097 (9)	0.0065 (9)	0.0155 (10)
C11	0.0401 (13)	0.0523 (15)	0.0461 (13)	0.0114 (11)	0.0151 (10)	0.0206 (11)
C12	0.0440 (14)	0.0568 (16)	0.0663 (16)	0.0077 (12)	0.0170 (12)	0.0356 (14)
C13	0.0479 (15)	0.0392 (14)	0.0766 (17)	0.0115 (11)	0.0190 (13)	0.0263 (13)
C14	0.0419 (13)	0.0437 (14)	0.0487 (13)	0.0107 (11)	0.0134 (11)	0.0124 (11)
C15	0.0322 (12)	0.0388 (12)	0.0428 (12)	0.0104 (10)	0.0084 (10)	0.0132 (10)
C16	0.0387 (13)	0.0433 (14)	0.0515 (13)	0.0124 (11)	0.0027 (11)	0.0135 (11)
C17	0.0431 (15)	0.0404 (14)	0.0751 (17)	0.0103 (12)	−0.0045 (13)	0.0104 (13)
C18	0.0405 (14)	0.0442 (15)	0.0562 (14)	0.0145 (11)	0.0076 (12)	0.0146 (12)
C19	0.0451 (15)	0.0602 (16)	0.0596 (15)	0.0165 (12)	0.0001 (12)	0.0269 (13)
C20	0.071 (2)	0.0528 (18)	0.143 (3)	0.0068 (16)	0.003 (2)	0.045 (2)
C21	0.076 (2)	0.0606 (18)	0.078 (2)	0.0289 (16)	−0.0061 (16)	−0.0043 (16)
N1	0.0396 (11)	0.0438 (11)	0.0437 (10)	0.0062 (9)	0.0088 (8)	0.0232 (9)
O1	0.0455 (11)	0.0551 (11)	0.0751 (12)	0.0121 (9)	−0.0145 (9)	0.0129 (10)
O2	0.0498 (11)	0.0427 (10)	0.0749 (11)	0.0169 (8)	−0.0066 (9)	0.0132 (9)
O3	0.0483 (11)	0.0482 (10)	0.0695 (11)	0.0112 (8)	−0.0148 (9)	0.0091 (9)
S1	0.0433 (4)	0.0417 (4)	0.0459 (3)	0.0025 (3)	0.0043 (3)	0.0141 (3)
S2	0.0507 (4)	0.0436 (4)	0.0557 (4)	0.0122 (3)	0.0204 (3)	0.0243 (3)

### Geometric parameters (Å, º)

**Table:**

C1—C2	1.391 (3)	C12—C13	1.379 (3)
C1—C6	1.403 (3)	C12—H12	0.9300
C1—S1	1.729 (2)	C13—C14	1.378 (3)
C2—C3	1.367 (3)	C13—H13	0.9300
C2—H2	0.9300	C14—H14	0.9300
C3—C4	1.377 (3)	C15—C16	1.336 (3)
C3—H3	0.9300	C15—C18	1.471 (3)
C4—C5	1.377 (3)	C16—O3	1.334 (3)
C4—H4	0.9300	C16—C17	1.503 (3)
C5—C6	1.385 (3)	C17—O2	1.450 (3)
C5—H5	0.9300	C17—C20	1.517 (4)
C6—N1	1.397 (3)	C17—C21	1.524 (4)
C7—N1	1.289 (2)	C18—O1	1.201 (3)
C7—S2	1.741 (2)	C18—O2	1.360 (3)
C7—S1	1.754 (2)	C19—O3	1.433 (3)
C8—C9	1.504 (3)	C19—H19A	0.9600
C8—S2	1.820 (2)	C19—H19B	0.9600
C8—H8A	0.9700	C19—H19C	0.9600
C8—H8B	0.9700	C20—H20A	0.9600
C9—C14	1.387 (3)	C20—H20B	0.9600
C9—C10	1.406 (3)	C20—H20C	0.9600
C10—C11	1.390 (3)	C21—H21A	0.9600
C10—C15	1.480 (3)	C21—H21B	0.9600
C11—C12	1.378 (3)	C21—H21C	0.9600
C11—H11	0.9300		
			
C2—C1—C6	121.1 (2)	C13—C14—C9	121.5 (2)
C2—C1—S1	129.24 (18)	C13—C14—H14	119.3
C6—C1—S1	109.60 (16)	C9—C14—H14	119.3
C3—C2—C1	117.8 (2)	C16—C15—C18	105.53 (19)
C3—C2—H2	121.1	C16—C15—C10	134.4 (2)
C1—C2—H2	121.1	C18—C15—C10	120.01 (19)
C2—C3—C4	121.6 (2)	O3—C16—C15	133.6 (2)
C2—C3—H3	119.2	O3—C16—C17	114.12 (19)
C4—C3—H3	119.2	C15—C16—C17	112.3 (2)
C3—C4—C5	121.4 (2)	O2—C17—C16	102.18 (18)
C3—C4—H4	119.3	O2—C17—C20	107.9 (2)
C5—C4—H4	119.3	C16—C17—C20	112.3 (2)
C4—C5—C6	118.3 (2)	O2—C17—C21	107.9 (2)
C4—C5—H5	120.8	C16—C17—C21	112.4 (2)
C6—C5—H5	120.8	C20—C17—C21	113.3 (2)
C5—C6—N1	124.9 (2)	O1—C18—O2	120.9 (2)
C5—C6—C1	119.8 (2)	O1—C18—C15	129.0 (2)
N1—C6—C1	115.26 (18)	O2—C18—C15	110.17 (19)
N1—C7—S2	126.65 (17)	O3—C19—H19A	109.5
N1—C7—S1	116.90 (16)	O3—C19—H19B	109.5
S2—C7—S1	116.43 (12)	H19A—C19—H19B	109.5
C9—C8—S2	113.56 (14)	O3—C19—H19C	109.5
C9—C8—H8A	108.9	H19A—C19—H19C	109.5
S2—C8—H8A	108.9	H19B—C19—H19C	109.5
C9—C8—H8B	108.9	C17—C20—H20A	109.5
S2—C8—H8B	108.9	C17—C20—H20B	109.5
H8A—C8—H8B	107.7	H20A—C20—H20B	109.5
C14—C9—C10	118.79 (19)	C17—C20—H20C	109.5
C14—C9—C8	120.27 (18)	H20A—C20—H20C	109.5
C10—C9—C8	120.93 (18)	H20B—C20—H20C	109.5
C11—C10—C9	118.73 (19)	C17—C21—H21A	109.5
C11—C10—C15	119.70 (18)	C17—C21—H21B	109.5
C9—C10—C15	121.48 (18)	H21A—C21—H21B	109.5
C12—C11—C10	121.7 (2)	C17—C21—H21C	109.5
C12—C11—H11	119.2	H21A—C21—H21C	109.5
C10—C11—H11	119.2	H21B—C21—H21C	109.5
C11—C12—C13	119.5 (2)	C7—N1—C6	109.71 (18)
C11—C12—H12	120.3	C18—O2—C17	109.83 (17)
C13—C12—H12	120.3	C16—O3—C19	119.67 (17)
C14—C13—C12	119.9 (2)	C1—S1—C7	88.52 (10)
C14—C13—H13	120.1	C7—S2—C8	101.71 (10)
C12—C13—H13	120.1		
			
C6—C1—C2—C3	−0.3 (3)	C18—C15—C16—C17	−1.3 (3)
S1—C1—C2—C3	177.37 (19)	C10—C15—C16—C17	175.3 (2)
C1—C2—C3—C4	0.2 (4)	O3—C16—C17—O2	−178.22 (19)
C2—C3—C4—C5	−0.4 (4)	C15—C16—C17—O2	1.5 (3)
C3—C4—C5—C6	0.7 (4)	O3—C16—C17—C20	−62.8 (3)
C4—C5—C6—N1	−178.7 (2)	C15—C16—C17—C20	117.0 (3)
C4—C5—C6—C1	−0.8 (3)	O3—C16—C17—C21	66.4 (3)
C2—C1—C6—C5	0.6 (3)	C15—C16—C17—C21	−113.8 (2)
S1—C1—C6—C5	−177.46 (16)	C16—C15—C18—O1	−179.5 (2)
C2—C1—C6—N1	178.74 (18)	C10—C15—C18—O1	3.3 (4)
S1—C1—C6—N1	0.6 (2)	C16—C15—C18—O2	0.5 (2)
S2—C8—C9—C14	114.53 (19)	C10—C15—C18—O2	−176.70 (18)
S2—C8—C9—C10	−64.9 (2)	S2—C7—N1—C6	−177.70 (14)
C14—C9—C10—C11	−0.3 (3)	S1—C7—N1—C6	0.6 (2)
C8—C9—C10—C11	179.14 (18)	C5—C6—N1—C7	177.21 (19)
C14—C9—C10—C15	176.32 (18)	C1—C6—N1—C7	−0.8 (2)
C8—C9—C10—C15	−4.2 (3)	O1—C18—O2—C17	−179.5 (2)
C9—C10—C11—C12	0.4 (3)	C15—C18—O2—C17	0.5 (3)
C15—C10—C11—C12	−176.34 (19)	C16—C17—O2—C18	−1.1 (3)
C10—C11—C12—C13	0.0 (3)	C20—C17—O2—C18	−119.7 (2)
C11—C12—C13—C14	−0.4 (3)	C21—C17—O2—C18	117.5 (2)
C12—C13—C14—C9	0.4 (3)	C15—C16—O3—C19	−4.9 (4)
C10—C9—C14—C13	−0.1 (3)	C17—C16—O3—C19	174.8 (2)
C8—C9—C14—C13	−179.52 (19)	C2—C1—S1—C7	−178.2 (2)
C11—C10—C15—C16	−71.7 (3)	C6—C1—S1—C7	−0.26 (14)
C9—C10—C15—C16	111.7 (3)	N1—C7—S1—C1	−0.19 (15)
C11—C10—C15—C18	104.6 (2)	S2—C7—S1—C1	178.27 (12)
C9—C10—C15—C18	−72.1 (3)	N1—C7—S2—C8	−6.61 (19)
C18—C15—C16—O3	178.4 (2)	S1—C7—S2—C8	175.11 (10)
C10—C15—C16—O3	−5.0 (4)	C9—C8—S2—C7	−80.97 (16)

### Hydrogen-bond geometry (Å, º)

**Table:**

*D*—H···*A*	*D*—H	H···*A*	*D*···*A*	*D*—H···*A*
C2—H2···O1^i^	0.93	2.56	3.302 (3)	137

Symmetry code: (i) *x*−1, *y*, *z*.
